# The Hot Water Cure

**Published:** 1883-10

**Authors:** 


### THE HOT WATER CURE.

IT is remarkable how an old idea may be "revamped," burnished up, and made to pass for new. Reader! you may have had a mother; not the woman who considered her duty to God, to herself, to you and to society finished when she gave you birth, and then consigned you to Biddy, or Dinah to be carried off to the nursery—that tomb of "mother love," whose gloom can never be dispelled by sunshine nor all the trappings of luxury—but a real mother who blended her life with yours, anticipating your wants and ever watchful of ills that might overtake you. And if you do not remember, you have heard how she cured you of colic with warm herb teas, and hot draughts to your feet; she cured croup by dipping strips of flannel in hot water, then wringing them out and enveloping your neck with them; how she cured a cold and cough by wetting several thicknesses of flannel in hot water and laying them on your chest. But the world has forgotten its experiences, and HOT WATER poses as a brand-new remedy; not only for ailments for which it is especially adapted, but is recommended by some who ought to know better for diseases that where it might do positive harm.

Do not imagine that because water is abundant, is found everywhere, even in stones and metals, that it has no potency as a curative agent. It stands at the head of the list of remedies, and enters into all compounds. It constitutes five-sixths of the material from which the bodies of men and animals are made. A knowledge of these facts will enable us to see more clearly how water, and particularly hot water, acts as a remedial agent. Take, for example, the case of a person who has taken cold in the lungs. The circulation of the blood in the small blood vessels in that portion of the lungs affected becomes sluggish; in some cases it is quite suspended; the general circulation is impeded through failure of an important organ to do the work required of it, and the whole system suffers; the man is ill. Now, if we know why the disease exists, by what unnatural condition it is kept up, the remedy suggests itself; as, if a water pipe were frozen up, any child knows that the remedy is heat. And here is just where water as warm as it can be comfortably borne will effect a cure in ordinary cases. Let the patient go to bed. Put bottles of *hot water* to his feet, and cloths wet in *hot water* on his chest. Let him drink *hot water* as freely as he can with comfort; it matters little whether it is clear hot water, or herb tea, it is nevertheless *hot water*. With this treatment we are employing hot water at its full value. Its internal use tends to *thaw out* the blood vessels, and its outward application quickens the circulation in the blood vessels near the surface; thus drawing on the deep-seated blood vessels for supplies to keep up the activity, and thus the congestion is relieved and the patient is cured.

In dyspepsia, hot water, taken internally, under proper restrictions, is no doubt very useful, since dyspepsia depends on a congested and deranged condition of the digestive organs. But in consumption and other diseases attended by general debility it can only be detrimental. When a person is feeble from disease not marked with acute inflammation, the hot water treatment necessarily increases the debility. Here a tonic treatment is applicable—a treatment that will increase and enrich the blood and supply the fuel required to keep the machinery of life in motion. The Hot Water Treatment is useful in removing obstructions from the machinery, but only in systems where there is a surplus of vital power.

To recapitulate: the drinking of hot water at proper intervals and in proper quantities is useful in dyspepsia, constipation, torpid liver, congestion of the stomach, chronic diarrhœa, and in various affections of the kidneys and bladder ; provided that there is not at the same time serious diseases of the lungs, with debility.

The water should be as hot as tea is usually madethat,—is from 110° to 150°, and should be sipped, not taken rapidly. The quantity should be from half a pint to a pint. It should be taken one to two hours after meals, and nothing should be eaten until at least one hour afterward. The evening draught should be just before going to bed. The hot water treatment should continue until a cure is effected ; the time required will vary from one to six months.

If a person desires to reduce this treatment to a system, and thus be able to judge for himself of the required temperature of the water, its quantity, and how often it should be taken, he should provide himself with a *thermometer* and a *urinometer.* The thermometer will enable him to get the proper temperature of the hot water, and the urinometer will show the specific gravity of the urine that is voided on rising in the morning ; this being the proper time to make the test.

The specific gravity of the urine of a healthy infant is 1015 to 1020. The near approach to these figures indicates that the treatment is properly regulated. The urine should be near the color of champagne, free from rank odor and of sediment on cooling.

In order to derive the full benefit of this, or indeed of any treatment, the diet and the general habits of the patient should be regulated to conform with the treatment. All excesses in eating and drinking should be avoided.

The effect of the above treatment, properly carried out, is to improve the general health by removing obstructions to the natural action of the digestive organs, upon which the condition of the other internal organs mainly depends.

